# Enhanced UV Emission from ZnO on Silver Nanoparticle Arrays by the Surface Plasmon Resonance Effect

**DOI:** 10.1186/s11671-020-03470-2

**Published:** 2021-01-07

**Authors:** Xiao Wang, Qiong Ye, Li-Hua Bai, Xi Su, Ting-Ting Wang, Tao-Wei Peng, Xiao-Qi Zhai, Yi Huo, Hao Wu, Chang Liu, Yu-Yu Bu, Xiao-Hua Ma, Yue Hao, Jin-Ping Ao

**Affiliations:** 1 National Key Discipline Laboratory of Wide Band-Gap Semiconductor, School of Microelectronics, Xidian University, Xi’an, 710071 People’s Republic of China; 2 School of Science, Xi’an Shiyou University, Xi’an, 710065 People’s Republic of China; 3 Key Laboratory of Artificial Micro- and Nanostructures of Ministry of Education, School of Physics and Technology, Wuhan University, Wuhan, 430072 People’s Republic of China

**Keywords:** Surface plasmons, Zinc oxide, Silver nanoparticle arrays, Anodic aluminum oxide templates

## Abstract

Periodical silver nanoparticle (NP) arrays were fabricated by magnetron sputtering method with anodic aluminum oxide templates to enhance the UV light emission from ZnO by the surface plasmon resonance effect. Theoretical simulations indicated that the surface plasmon resonance wavelength depended on the diameter and space of Ag NP arrays. By introducing Ag NP arrays with the diameter of 40 nm and space of 100 nm, the photoluminescence intensity of the near band-edge emission from ZnO was twofold enhanced. Time-resolved photoluminescence measurement and energy band analysis indicated that the UV light emission enhancement was attributed to the coupling between the surface plasmons in Ag NP arrays and the excitons in ZnO with the improved spontaneous emission rate and enhanced local electromagnetic fields.

## Introduction

Recently, surface plasmons (SPs) have attracted much attention. In particular, as the collective oscillations of free electrons around metal nanoparticles (NPs) surface, localized surface plasmons (LSPs) were widely applied to enhance the light emission in optoelectronic devices, due to selective photon absorptions and enhanced local electromagnetic field around the metal NPs [1]. Many efforts of LSP-enhanced emissions have been made in ultraviolet (UV) optoelectronic devices such as light emitting diodes [2–4] and photodetectors [5–9].

ZnO is one of the most promising materials for UV optoelectronic devices due to a direct wide bandgap of 3.37 eV and an exciton binding energy of 60 meV [10]. However, the low UV light emission efficiency blocks its commercial application. Hence, different metals (Ag [11–18], Au [12, 15, 19, 20], Al [21–24], Cu [25], Ti [26, 27], Ni [27], Pt [28]) with different shapes (gratings, sphere, cylinder, triangular prism, tetragonal prism, bowtie) have been applied to enhance the near band-edge UV emission of ZnO. Among them, Ag NPs with a sphere shape composited by hydrothermal method were most widely used due to relatively easy fabrication and effective light emission enhancement. However, hydrothermally composited Ag NPs are usually randomly distributed, and it is hard to control the local electromagnetic fields distribution and wafer homogeneity. Therefore, electron beam lithography (EBL) and nanoimprint lithography were applied to obtain controllable shapes and arrangements. Nevertheless, expensive facility and difficult in large-scale manufacturing block the applications of the EBL and nanoimprint lithography [24].

In this work, the LSP-enhanced UV light emissions from ZnO were obtained by introducing periodical Ag NPs arrays with AAO templates. The optimal size of Ag NP arrays was obtained as the diameter of 40 nm and space of 100 nm with a twofold photoluminescence enhancement in the UV light emission of ZnO. The simulation and experimental photoluminescence spectra were analyzed to reveal the mechanism of light emission enhancement.

## Methods

**Fig. 1:**
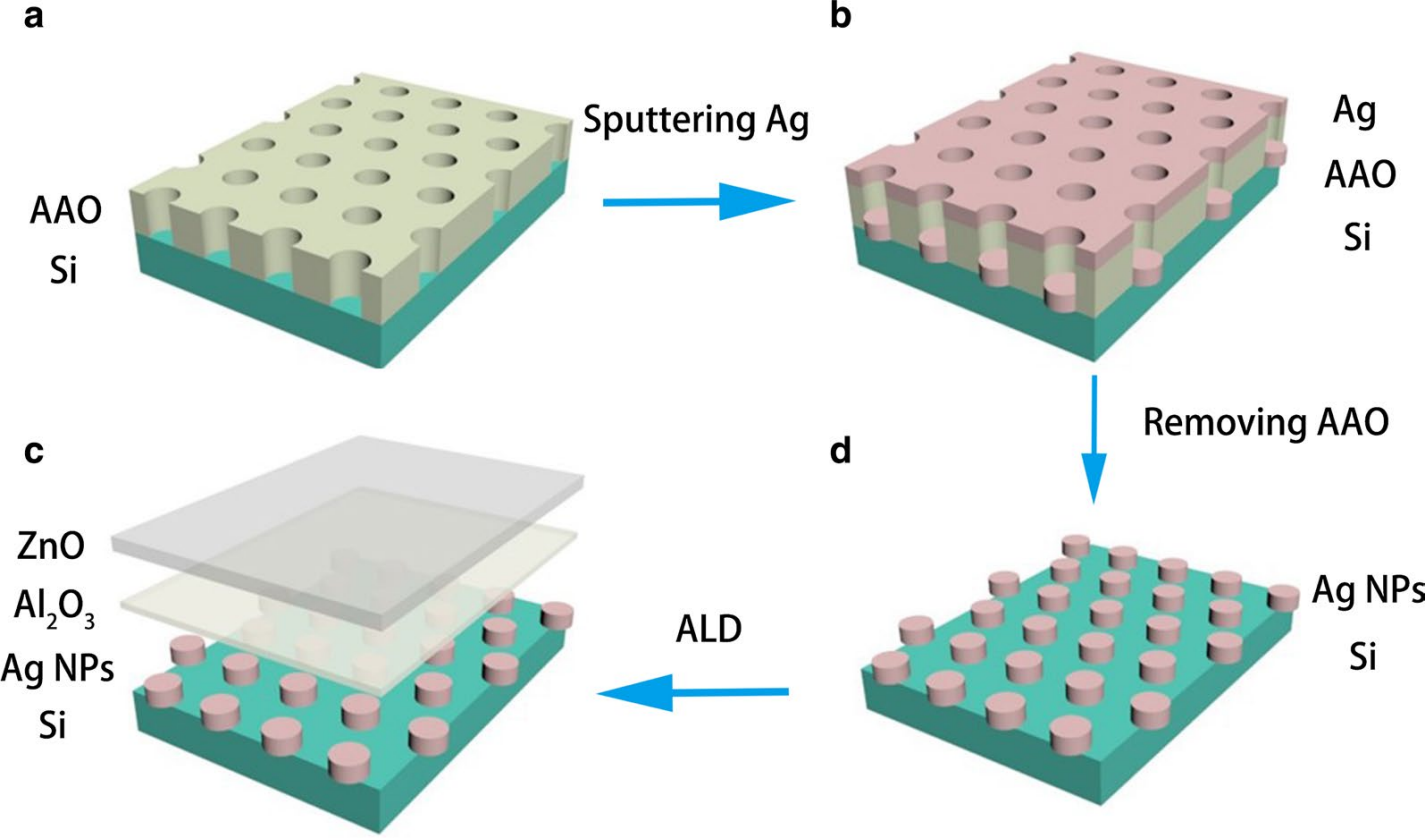
Fabrication process: **a** transferred AAO template on Si substrate, **b** magnetron sputtered Ag on AAO template, **c** remained silver arrays on Si substrate, **d** ZnO and Al_2_O_3_ on Ag NP arrays deposited by atomic layer deposition

The fabrication process is shown in Fig. 1. Firstly, commercial AAO templates were transferred on silicon substrate. The thickness of AAO templates is 200 nm with the diameter and space shown in Table 1. Sample 1 was fabricated without AAO templates and corresponding Ag NP arrays. Secondly, an Ag (35 nm) layer was deposited by magnetron sputtering with DC power of 100 W, pressure of 3 mTorr and Ar flow rate of 18 sccm. Thirdly, the AAO templates were removed by Kapton tape and Ag NP arrays were left on Si substrate. Finally, Al_2_O_3_ (10 nm) and ZnO (70 nm) films were in-turn grown on Ag NP arrays at 150 °C by atomic layer deposition (ALD), with trimethylaluminum (TMA), H_2_O and diethyl zinc (DEZn) as the sources of aluminum, oxygen and zinc, respectively. The growth details and characteristics of ZnO films can be found in our previous publication [29].

**Table 1:** The diameters and spaces of samples

Samples	Diameter (nm)	Space (nm)
1	–	–
2	30	65
3	40	65
4	40	100
5	60	100
6	70	125

The morphologies of AAO templates and Ag NP arrays were characterized by scanning electron microscope (SEM). The photoluminescence (PL) measurement was performed with a He-Cd laser ($\lambda$ = 325 nm). The time-resolved PL spectra were measured with excitation wavelength at 310 nm at room temperature to evaluate the light emission mechanism.

## Results and Discussion

**Fig. 2:**
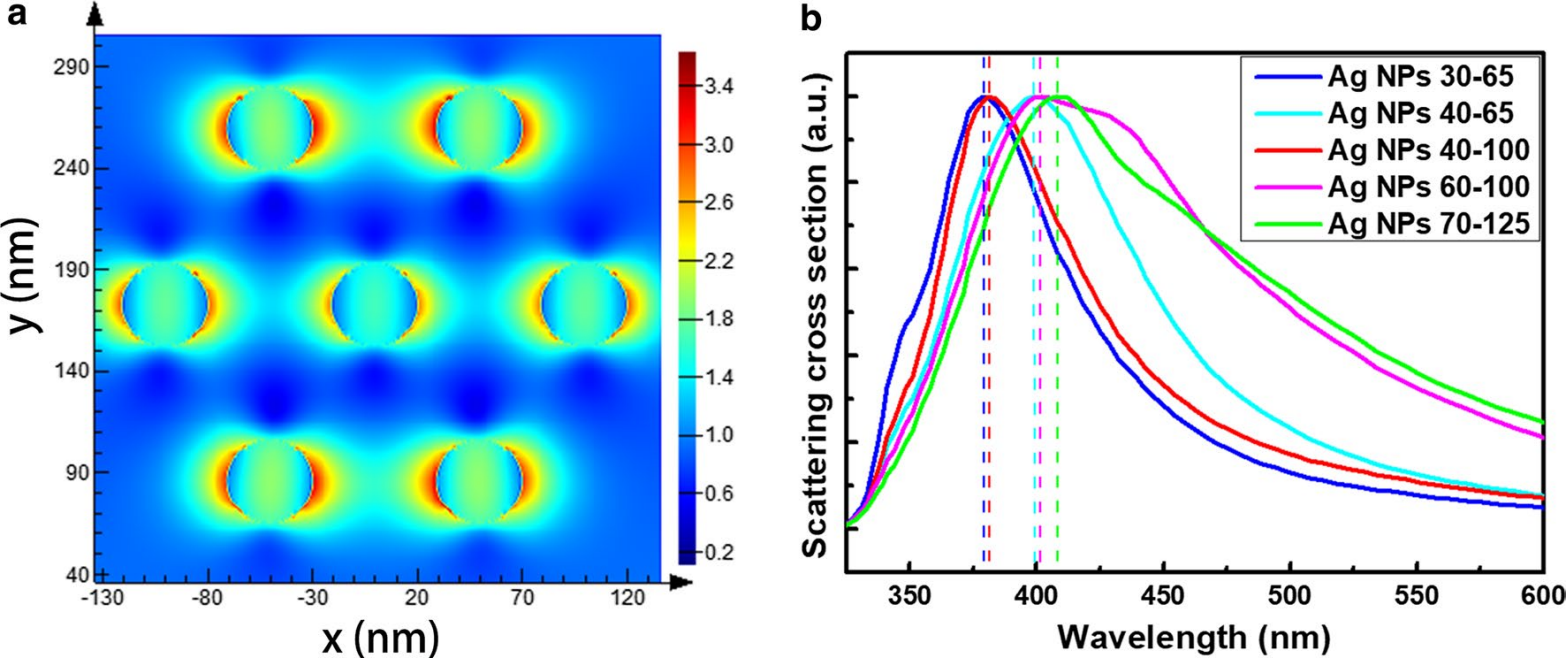
**a** Simulated spatial electric field distribution in the sample 4 with the diameter of 40 nm and space of 100 nm. **b** Normalized scattering cross section spectra of Ag NP arrays with different diameters and spaces

Before conducting the experiments, numerical calculations by the finite-difference time-domain (FDTD) method were performed to analyze the effect of different diameters and spaces of Ag NP arrays on the electric field distribution and surface plasmon resonance wavelengths. The electric field distributions and the scattering cross-section (*Q*_scat_) spectra of Ag NP arrays were simulated under a total-field scattered-field (TFSF) light source polarized along the *z*-axis. The analysis group was placed outside of the optical source to monitor the scattering cross section of light. The optical parameters of Ag were selected as the CRC model from the material database of the Lumerical FDTD solutions software. The simulated spatial electric field distribution in the sample 4 with the diameter of 40 nm and space of 100 nm is shown in Fig. 2a. The local electromagnetic field around the Ag NPs was enhanced about 3.5 times, leading to a strong coupling between the excitons in ZnO films and the SPs in Ag NP arrays, and resulting the light emission enhancement. Figure 2b shows the normalized *Q*_scat_ spectra of Ag NP arrays with different diameters and spaces. The surface plasmon resonance wavelengths of Ag NP arrays from sample 2 to sample 6 are 379, 399, 381, 402 and 408 nm, respectively. Considering the NBE emission of ZnO films around 383 nm, the optimized diameter and space of Ag NP arrays should be 40 and 100 nm in sample 4. By increasing the size of Ag NP arrays from sample 2 to 3, or from sample 4 to 5, the surface plasmon resonance wavelength makes a redshift under the same space condition. By increasing the space of Ag NP arrays from sample 3 to 4, the surface plasmon resonance wavelength makes a blueshift under the same diameter condition. Hence, the surface plasmon resonance wavelength of the Ag NP arrays depends on both the diameter and space of Ag NP arrays.

**Fig. 3:**
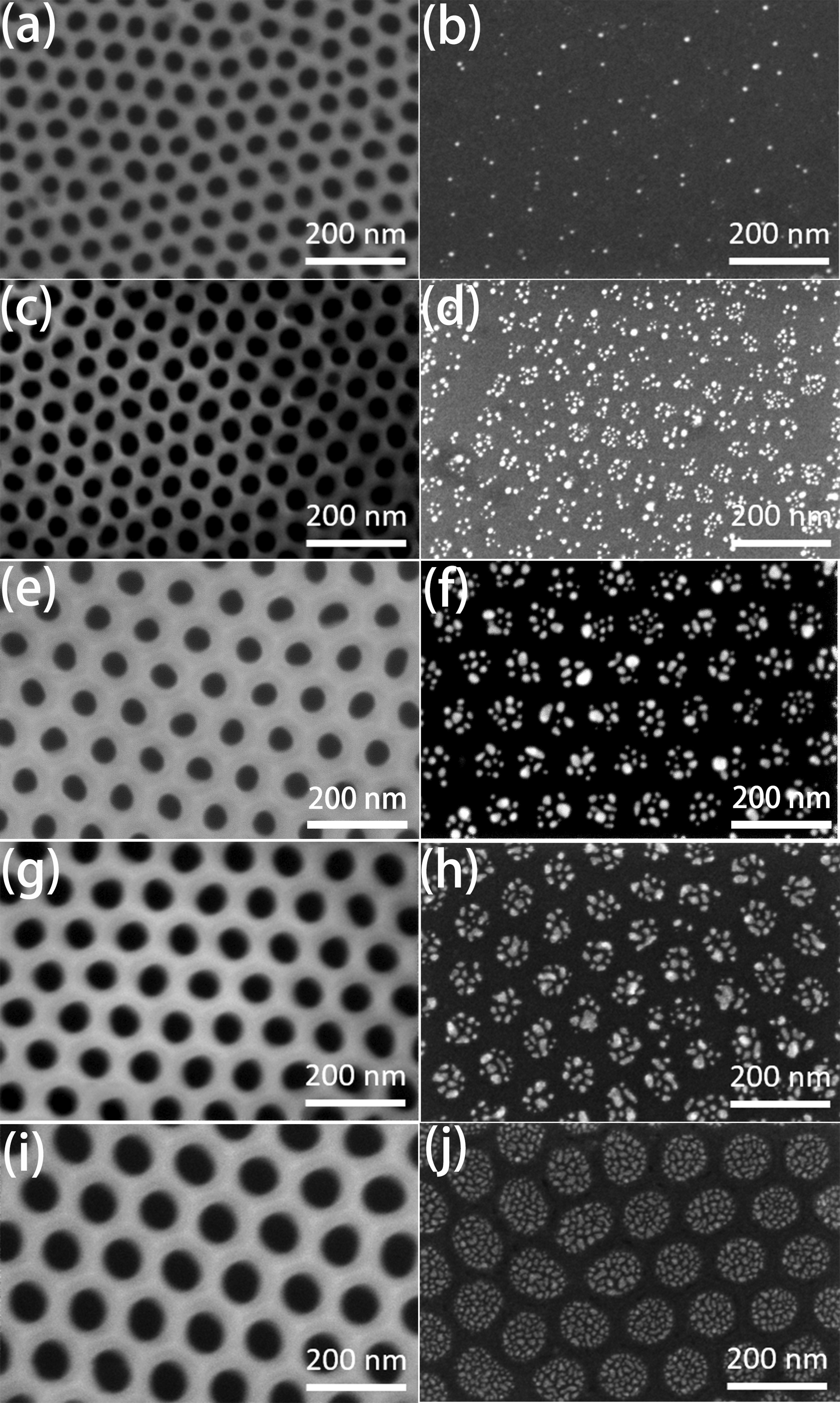
SEM images of transferred AAO templates of **a** sample 2, **c** sample 3, **e** sample 4, **g** sample 5, **i** sample 6, and corresponding Ag NP arrays of **b** sample 2, **d** sample 3, **f** sample 4, **h** sample 5, **j** sample 6

In Fig. 3, the transferred AAO templates and corresponding Ag NP arrays are demonstrated in the SEM images. As shown in Fig. 3a, c, e, g, i, the average diameters of AAO templates from sample 2 to sample 6 are measured to be 33, 38, 40, 61 and 71 nm, and the corresponding average spaces of AAO templates are 63, 61, 100, 101, 124 nm, which is in accord with the designed diameters and spaces in Table 1. As shown in Fig. 3b, d, f, h, j, the average diameters of Ag NP arrays from sample 2 to sample 6 are measured to be 8, 37, 46, 64 and 79 nm, and the corresponding average spaces of Ag NP arrays are 59, 62, 99, 102, 122 nm. When the diameter of AAO template is as small as 33 nm, it is hard to form periodic Ag NP arrays. As for the diameters of AAO templates ranging from 40 to 60 nm in sample 3, 4 and 5, the diameters of Ag NP arrays are in accord with these of AAO templates. When the diameter of AAO template is as big as 71 nm, the diameter of sputtered Ag NP arrays is slightly bigger than that of AAO template, which may be on account of the Ag NP dispersion at the removing of the Kapton tape. Generally, the measured Ag NP array spaces are well matched with the AAO template spaces and in accord with the designed sizes. And the obtained periodic Ag NP arrays can be accurately controlled by applying the corresponding AAO templates.

**Fig. 4:**
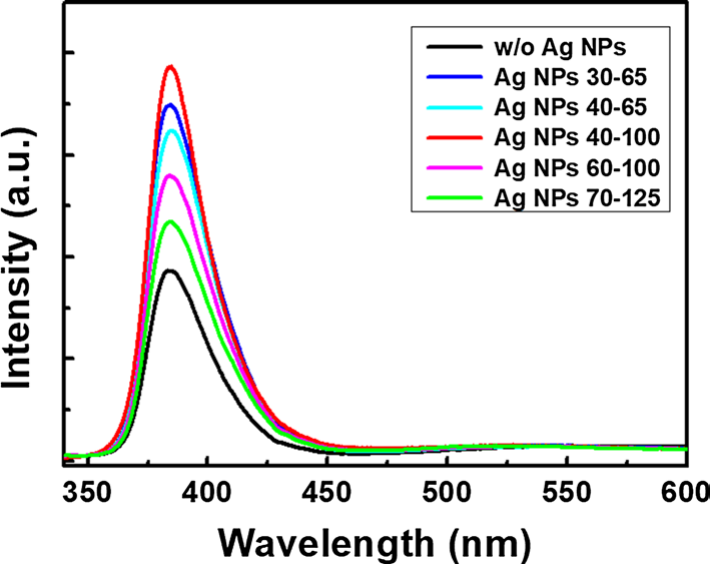
PL spectra of samples with different diameters and spaces at room temperature

Figure 4 presents the PL spectra of different samples at room temperature. As shown, a dominant peak at 383 nm and a weak peak at around 520 nm are obtained in the PL spectra, which are attributed to the near band-edge (NBE) emission and deep-level emission of ZnO, respectively. The deep-level emission was attributed to the oxygen vacancies [29]. The intensity ratio between the NBE peak and the deep-level peak was calculated to be 14 in the sample 1 without Ag NP arrays, indicating that ZnO films grown by ALD are in a good quality. The NBE peak intensities with Ag NP arrays are higher than that without Ag NP arrays, which is attributed to the coupling between the excitons in ZnO films and the SPs in Ag NP arrays, enhancing the local electromagnetic fields and increasing the spontaneous emission rate of ZnO. Among the PL curves of samples with different diameters and spaces, the NBE peak intensity in the sample 4 with the diameter of 40 nm and space of 100 nm is the strongest, which is twofold larger than that of sample 1 without Ag NP arrays, indicating that Ag NP arrays with the diameter of 40 nm and space of 100 nm are the optimum to enhance the light emission of ZnO, which is in accord with the simulation results above. Besides, the deep-level peak at around 520 nm was almost the same for all samples, leading to a high intensity ratio of 28 between the NBE peak and the deep-level peak in the sample 4.

**Fig. 5:**
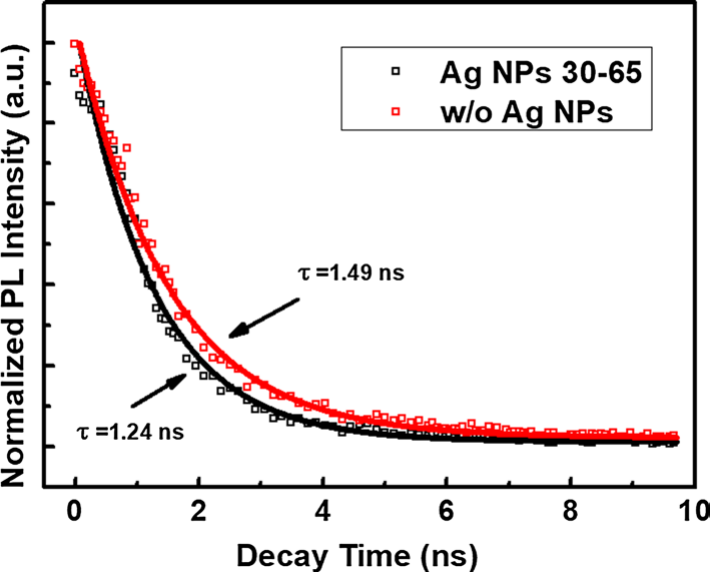
Time-resolved PL decays of sample 1 without Ag NP arrays and sample 2 with Ag NP arrays at 380 nm

To further analyze the mechanism of the enhanced UV light emission by adding Ag NP arrays, the time-resolved PL decays of sample 1 without Ag NP arrays and sample 2 with Ag NP arrays were performed at room temperature in Fig. 5. The decay curves are fitted with an exponential decay model to obtain the decay lifetimes (*τ*) with the equation $I\left(t\right)={I}_{0}\mathrm{exp}(-t/\tau )$. The decay lifetimes of sample 1 without Ag NP arrays and sample 2 with Ag NP arrays are deduced to be 1.49 and 1.24 ns, respectively. The reduced decay lifetime from 1.49 to 1.24 ns indicates a faster decay process in the ZnO with Ag NP arrays, which may attribute to the improved spontaneous emission rate by adding Ag NP arrays, enhancing the coupling between the SPs in Ag NP arrays and the excitons in ZnO.

**Fig. 6:**
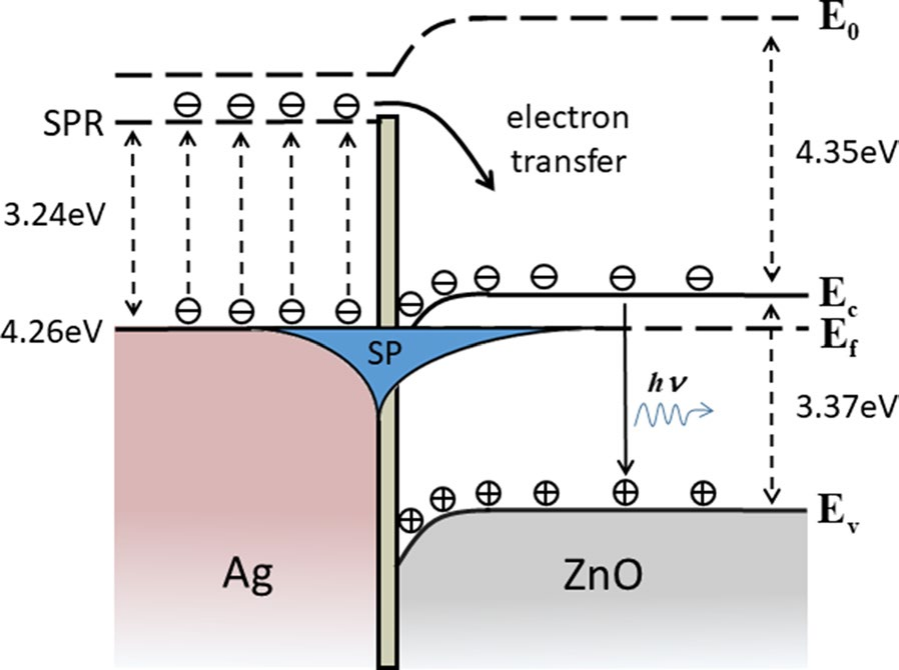
Schematic energy band diagram of Ag/Al_2_O_3_/ZnO structure

To further explain the improvement of the UV light emission, the energy band diagram of the Ag/Al_2_O_3_/ZnO structure is shown in Fig. 6. The work function of Ag is 4.26 eV and the electron affinity of ZnO is 4.35 eV, leading to a conduction band downward bending of ZnO near the Al_2_O_3_/ZnO interface. The 10-nm Al_2_O_3_ layer was applied to block Fӧrster-type non-radiative energy transfer process from semiconductor to metal [28]. Due to the surface plasmons generated between the metal and dielectric medium at the Ag/Al_2_O_3_ interface, the local electric field near Ag NP arrays is enhanced, increasing the excitation energy density of incident light and the number of absorbed photons within the coupling distance. Simultaneously, the enhanced local electric field also promotes the surface plasmons of Ag NPs coupling with excitons of ZnO, which will improve the spontaneous emission rate and enhance the photoluminescence intensity of ZnO. Besides, there may be another process that electrons in the Ag NP arrays jump to the SPR level and then transfer to the conduction band of the ZnO [28]. And the increased electron density in the conduction band will also enhance the NBE emission of ZnO.

## Conclusions

In summary, periodical Ag NP arrays were fabricated by magnetron sputtering method with AAO templates to enhance the UV light emission from ZnO by the surface plasmon resonance effect. Theoretical simulations indicated that the surface plasmon resonance wavelength depended on both the diameter and space of Ag NP arrays. By introducing Ag NP arrays with the diameter of 40 nm and space of 100 nm, the photoluminescence intensity of the near band-edge emission from ZnO was twofold enhanced. Time-resolved photoluminescence measurement and energy band analysis revealed that the improvement of UV light emission was attributed to the coupling between the SPs in Ag NP arrays and the excitons in ZnO with the improved spontaneous emission rate and enhanced local electromagnetic fields.

## Data Availability

The experiment data supporting the conclusion of this manuscript have been given in this manuscript. All data are fully available without restriction.

## Abbreviations

NP: Nanoparticle
AAO: Anodic aluminum oxide
SPs: Surface plasmons
LSPs: Localized surface plasmons
UV: Ultraviolet
ALD: Atomic layer deposition
SEM: Scanning electron microscope
PL: Photoluminescence
FDTD: Finite-difference time domain
